# Facilitated hospital-to-pre-hospital feedback for professional development (PHEM Feedback): a service evaluation using a self-reported questionnaire to understand the experiences of participating pre-hospital clinicians in the first year of operation

**DOI:** 10.29045/14784726.2023.6.8.1.42

**Published:** 2023-06-01

**Authors:** Matthew Snowsill, Gioacchino Cracolici, Talia Wieder, Grace Allen

**Affiliations:** St Mary’s Hospital ORCID iD: https://orcid.org/0000-0002-1276-4935; Cambridge University Hospitals NHS Foundation Trust ORCID iD: https://orcid.org/0009-0000-3780-0376; Whittington Health NHS Trust; London Ambulance Service

**Keywords:** education, feedback, mentors, paramedic

## Abstract

**Background:**

Information governance and resource challenges can impede pre-hospital clinicians from accessing and reflecting upon clinical information from the hospital phase of care, to ascertain how appropriate their diagnoses and management were. The authors performed a 12-month service evaluation of a hospital-to-pre-hospital feedback system, in which clinical information was requested by pre-hospital clinicians, and returned by a small team of hospital-based clinicians, while meeting information governance standards.

**Method:**

Pre-hospital clinicians in one ambulance station and one air ambulance service accessed patient information from a hospital, via a mediating senior pre-hospital colleague (a facilitator). Case-based learning conversations between the facilitator and clinician followed, using a report from the hospital. Evidence of benefit to the pre-hospital clinicians was prospectively collected using Likert-type scales, regarding general satisfaction, likelihood to change practice and effects on well-being. Reports aimed to be generated by the hospital within 14 days.

**Results:**

All 59 appropriate requests had reports returned. Of the reports, 59.5% were returned in 14 days or less. The median duration was 11 days (interquartile range 7–25). Learning conversations were completed in 86.4% (n = 51) of these cases, and of those, clinician questionnaires were completed in 66.7% (n = 34). Of the 34 questionnaire respondents, 82.4% (n = 28) were very satisfied with the returned information. A total of 61.1% (n = 21) were either likely or very likely to change their practice following the hospital’s information, and 64.7% (n = 22) reported similar or very similar impressions to the hospital’s eventual diagnosis. Regarding mental health, 76.5% (n = 26) reported positively or very positively affected mental health, while 2.9% (n = 1) reported adversely affected mental health. All of the respondents, 100% (n = 34) were either satisfied or very satisfied with the learning conversation.

**Conclusion:**

While hospital-based clinical information was successfully and securely provided to pre-hospital clinicians, these pilot data suggest it is not possible to meet the self-imposed, empirical 14-day target with four to five voluntary doctors. Sustained performance may improve with allocated or paid time to report the requests. The validity of these data is limited by a poor response rate, a non-validated questionnaire and potential for selection bias. Validation using multiple hospitals and greater numbers is the appropriate next step. Responses suggest that this system identifies areas for improvement, reinforces good practice and improves the mental well-being of the participating clinicians.

## Introduction

There is a desire among many pre-hospital clinicians to receive patient outcomes from the in-hospital phase of care to augment their learning and enhance their professional development. A study comparing hospital and pre-hospital diagnoses showed a low inter-rater reliability kappa value of 0.6 (95% confidence interval (CI) 0.5–0.7) (Brichko et al., 2016). Audit and feedback has been noted in a Cochrane review to have ‘small but potentially important improvements in professional practice’ (Ivers et al., 2012). This review noted that this is particularly the case when there is a lower baseline performance, when feedback came from supervisors and if an explicit action plan was incorporated.

Hospital-to-pre-hospital feedback (HPHF) is challenging to formalise and embed in UK practice, due to resource constraints and information governance reasons. One study using a small sample of semi-structured interviews noted a ‘dearth of formally established feedback’ in anything other than ‘exceptional’ incidents, and also noted a tendency for ambulance clinicians to pursue feedback through informal measures (Eaton-Williams et al., 2020b). Despite the barriers, guidance from regulators like the General Medical Council and Health and Care Professions Council emphasises the importance of clinicians following up with patients and engaging in reflective practice (Academy of Medical Royal Colleges et al., 2018; General Medical Council, 2013; Health and Care Professions Council, 2014). Such feedback may be an important means to improve diagnostic accuracy.

HPHF can come from a large number of sources. A 2017 cross-sectional survey of Emergency Medical Services (EMS) professionals in the United States described sources including peers, hospital staff, EMS supervisors and medical directors (Cash et al., 2017). Several HPHF systems exist (or have existed), often in the context of specific conditions. Examples include for cardiac arrest (Bleijenberg et al., 2017; Brinkrolf et al., 2018; Hostler et al., 2011; Hubner et al., 2017; Kramer-Johansen et al., 2006; Lukas et al., 2012; Lyon et al., 2012), trauma (Scott et al., 2017), stroke (Choi et al., 2014) and myocardial infarction (Daudelin et al., 2010; Scholz et al., 2006; Siriwardena et al., 2014).

A literature review examining 15 observational studies recommended that HPHF be balanced, timely and delivered by an appropriate person (Eaton-Williams et al., 2020a). The Eaton-Williams et al. (2020a) review and a Canadian qualitative study by Morrison et al. (2017) also concluded that this work may contribute to well-being, particularly when creating a wider culture where feedback and constructive exploration of areas is widely accepted to improve practice.

There are few peer-reviewed publications from UK-based, non-condition-specific feedback systems. Some have been published in the form of posters and conference abstracts (Jenkinson et al., 2009; Pollard & Black, 2015; Snowsill, 2020; Sommers et al., 2019). Most of these systems are designed to transfer patient data directly between the hospital and the pre-hospital clinicians who attended the patient (Eaton-Williams et al., 2020a).

The authors designed an HPHF system called ‘PHEM Feedback’. PHEM (an acronym for ‘pre-hospital emergency medicine’) was used to encompass both the worlds of the Helicopter Emergency Medical Services (HEMS) and ambulance services. The aim was to provide pre-hospital clinicians with legitimate, secure, reliable and proportionate access to a hospital’s information regarding patients for whom they have cared. This was intended to promote shared learning, reinforce good practice, highlight areas for improvement, foster an organisational ‘learning culture’ and support the psychological well-being of pre-hospital staff. Patients could opt out. This process satisfied national data-sharing standards. Support from the Health Research Authority’s Confidentiality Advisory Group (CAG) was granted on April 2018 to transfer patient information without consent as part of a non-research project. Support was also gained from local and national patient advocacy groups.

Pre-hospital clinicians obtained hospital information via an objective, senior member of their service (‘the facilitator’) who had not been involved in the case. It was their responsibility to assess the pre-hospital clinician’s request, ensure there was genuine clinical involvement, mutually agree learning objectives, submit the request to the hospital, receive the report back and then conduct a learning conversation (LC) in a psychologically safe manner. This ‘indirect’, facilitator-mediated model of feedback differed from other ‘direct’ feedback systems (i.e. ones conducted directly between the pre-hospital clinician and hospital) reported elsewhere (Pollard & Black, 2015; Sommers et al., 2019).

### Aims

1. Complete reports for 100% of appropriate requests within 14 days of the request being sent by the facilitator. Although non-evidence-based, this target was chosen prospectively on a pragmatic basis to balance the limitations of a small hospital team, while also trying to ensure timely access to outcomes for pre-hospital clinicians.
2. Evaluate the suitability of the 14-day target by examining any association between time to return the report and rate of pre-hospital clinician questionnaire completion.
3. Collect descriptive information to guide improvements to future standard operating procedures.
4. Measure the benefits as perceived by pre-hospital clinicians.

## Methods

Data collection for this prospective, single-centre project was undertaken over a 12-month period from 23 April 2018 to 22 April 2019. Staff from one ambulance station with approximately 110 clinical staff were eligible. The clinical managers (n = 7) acted as facilitators. Staff from a HEMS organisation (approximately 35 clinicians) were also eligible to participate. Approximately 100–120 HEMS patients were transported to the participating hospital that year (around 5% of their patients). Clinical HEMS staff were required to access HPHF via their ‘patient liaison managers’ (n = 2). A team from the district general hospital in Essex, United Kingdom (n = 9 over the study period with a peak of five staff at any one time) extracted clinical information when requested.

**Figure 1:**
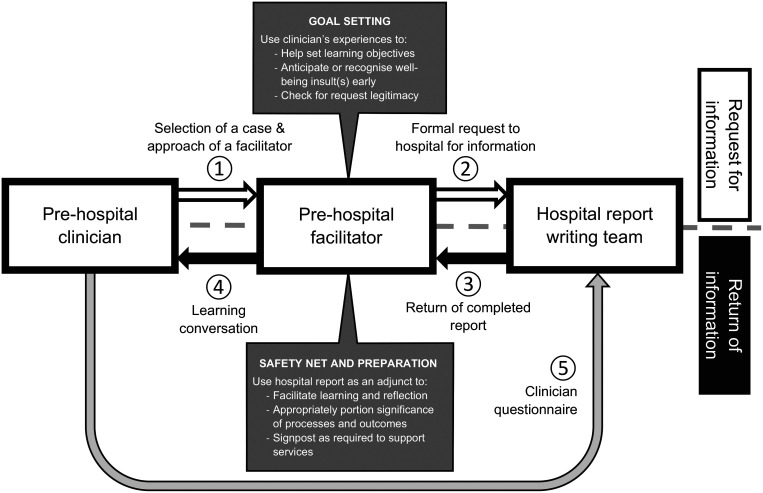
Figure 1. Stages of the information transfer between hospital and pre-hospital teams.

To learn what happened to their patients, ambulance service clinicians were required to approach a facilitator. The facilitators had agreed to review requests for legitimacy, educational merit and signs of potential psychological injury. It was the facilitator who submitted the request for information to the hospital team citing learning objectives, which they and the pre-hospital clinician had mutually agreed during a preliminary discussion. Potential information included diagnoses, functional condition, treatments, investigation results and details of clinical assessments. The facilitators also committed to receiving the report back before the pre-hospital clinicians, so that LCs could be planned prior to the pre-hospital clinicians learning the patient outcomes (Figure 1). This was to maximise educational value and allow for careful planning, in case patients went on to have adverse outcomes, whether the pre-hospital clinician was at fault or not. HEMS teams used the information within their existing clinical governance structures such as ‘death and disability’ meetings.

Hospital-based clinicians with no experience of pre-hospital care (nor the knowledge, skills and training pathways of those working for ambulance services or HEMS) wrote the reports. The hospital-based clinicians were not formally trained in giving feedback. They were expected to write the reports in a way which did not require in-depth knowledge of hospital practice like laboratory values and normal ranges. Opinions from hospital teams regarding pre-hospital management was actively discouraged. To enhance the effectiveness of the feedback, the facilitators were expected to be able to contextualise the hospital report and apply it to pre-hospital care, including identifying areas for future improvement, addressing further queries, identifying further learning resources and proposing further goals to aid the pre-hospital clinicians’ continuing professional development. The involvement of facilitators and the use of LCs was also designed to provide an opportunity for aftercare where patients had poor outcomes (regardless of blame) or distress persisted. A fuller description of the process is detailed in Supplementary 1.

Cases were eligible for analysis if the initial request was submitted within the 12-month period. For cases which had outstanding pre-hospital clinician questionnaires at the end of the 12 months, it was presumed that their LCs had not been completed if there was no questionnaire completed in the 30 days after facilitators’ requests.

Completion of the report, LC and questionnaire steps were binary (completed or not). Data were analysed to establish whether the time taken to complete a report altered the completion of questionnaires. The groups were separated into 14 days or less to return feedback from the hospital to the facilitator, and more than 14 days. Questionnaire completion rates were used as a surrogate for pre-hospital clinicians’ ongoing educational investment in the service and intended to examine whether engagement was lost after a certain length of time.

Statistics were analysed using Graphpad Prism 8 (Graphpad Software LLC). The rate of requests from pre-hospital teams is expressed as requests per month. The rates of report, debrief and questionnaire completion were analysed as percentages and absolute values. The duration between the receipt of a request and the sending of a report was measured in whole days, based on the difference between the respective dates. A median number of days and the interquartile range (IQR) were calculated. The numbers of dissenting patients and data breaches are presented as absolute values.

The authors noted generally longer times between requests and report completion in the latter 6-month period of the year. A post-hoc analysis was completed to statistically analyse the difference in completion of reports in less than or equal to 14 days between the first and second 6-month periods. This was analysed using a Mann-Whitney test to compare medians with a 95% CI.

Fisher’s exact test was applied to examine the association between completion of a report in 14 days or less and completion of the pre-hospital clinician questionnaire. A receiver operating characteristic (ROC) curve was used to determine the optimal balance which allowed for the greatest length of time for hospital teams to complete a report without negatively influencing the questionnaire response rate. The ROC curve analysis was performed using SPSS version 25 (IBM Corp, 2017). The true positive rate represents the proportion of returned questionnaires for which the report had been issued in the defined number of days or less, and the false positive rate is the proportion of unreturned questionnaires resulting from reports completed in the defined number of days or less.

The pre-hospital clinician questionnaire included five Likert-type scales (LTS) questions, each with five choices. Responding clinicians scored their overall satisfaction of the sufficiency and quality of the information returned by the hospital, the similarity between their and the hospital’s diagnoses, their likelihood to change their practice, the impact on their mental well-being and their satisfaction with the LC itself (Supplementary 2). The distribution of the answers was demonstrated by percentages. There were two open-answer questions on the survey regarding any further learning goals and general comments (Supplementary 3). Pre-hospital clinicians completed the questionnaires after their LC had concluded. Future access to the project was conditional on questionnaire completion, but responding was not incentivised in any other way.

The LTS responses were converted to a 5-point numerical scale (1 being most ‘negative’ and 5 being most ‘positive’). It is generally considered reasonable to perform this conversion but it is more appropriate when doing so to treat these scales as non-parametric data unless datasets are very large (Bishop & Herron, 2015; Harpe, 2015; Sullivan & Artino, 2013). The data were treated as non-parametric as the authors felt it could not be assumed that the results would follow a ‘normal distribution’. The LTS question datasets were analysed as absolute values and percentages.

### Ethics

Ethics approval was not required as this was a non-research service evaluation. However, careful consideration of the ethics surrounding the use of patient identifiable data (PID) was given. Any professional development for pre-hospital clinicians could only take place after the completion of the pre-hospital care episode, therefore the use of PID was considered of benefit to the clinicians, rather than directly to the patient themselves.

Consent-based models were considered and excluded by the project team. Challenges were anticipated with reliably and consistently consenting within several vulnerable patient cohorts, including advanced dementia, delirium, intoxication, exacerbations of mental health conditions, brain injuries, children or those who subsequently died. Excluding such patients from the process could lead to future patients being treated by pre-hospital clinicians who had missed the opportunity to learn more about the common care needs shared with others in these cohorts. The authors anticipated that consenting patients in the midst of being treated risked an imbalance of power (whether perceived or actual) to influence the tendency to consent, possibly from a sense of wanting to display gratitude even if they would prefer not to consent, and worry that dissenting could affect their standard of care. Patients might be understandably distracted by their acute health problem and unable to retain information or be truly, fully informed. The authors considered prospective, delayed consent. A review of the patients who attended the hospital via the emergency department (ED) in the prior year revealed that 97% of patients were no longer in the hospital 7 days after their ED presentation. It was felt that it was not practicable with the resources available to be able to consent enough of these patients. In cases where it would be possible, this would risk interrupting personal care, therapy interventions, meal times or other important in-patient activities. CAG deemed these considerations adequate to justify a dissent-based model. The standard operating procedures had been reviewed and supported by the hospital’s ‘Patient Panel’ of patient representatives and a national charity for health service users and disabled people as part of the CAG process.

Patients were informed of the activity with posters in patient-facing areas of the ED and information on the hospital website. This included instructions for opting out via email, written correspondence, telephone and engagement with the hospital’s patient advocacy groups.

## Results

A total of 59 requests were received by the hospital; 31 pre-hospital clinicians participated and seven facilitators participated during the data collection period (Supplementary 4). Two facilitators were responsible for initiating the majority of requests to the hospital team between them, 28 (47.5%) and 22 (37.3%) respectively. There were three inclusion criteria for requests: ‘significant diagnostic uncertainty’, ‘critically unwell patient’ and ‘significant emotional impact’. These were the reasons cited in 80.70% (n = 46), 59.65% (n = 65) and 1.75% (n = 1) of cases respectively.

**Figure 2:**
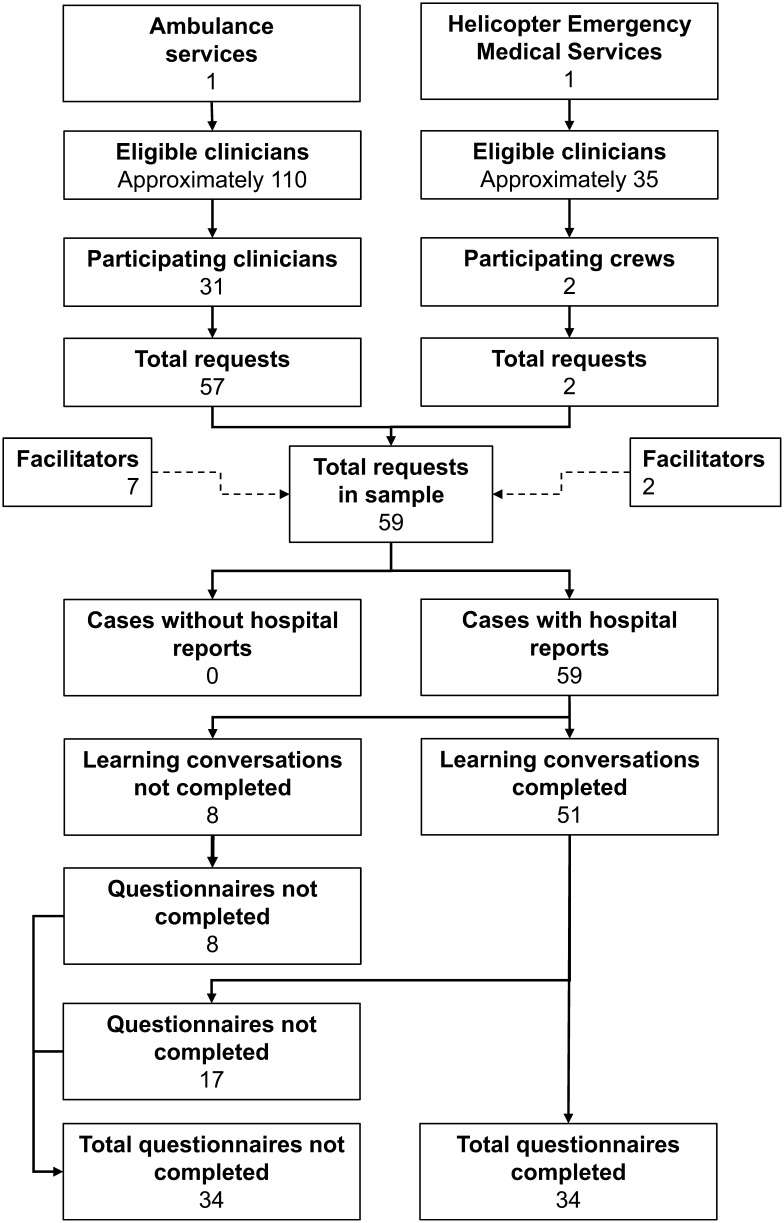
Figure 2. Staff participation and project performance in absolute numbers.

Reports were completed in 100% (n = 59) of requested cases (Figure 2); 59.5% (n = 37) were completed in 14 days or less. The median time was 11 days (IQR 7–25) (Table 1). Post-hoc analysis of the two 6-month periods showed a reduction in ‘14-day’ performance from 86.2% to 40.0%. The median time increased from 7 days (IQR 3–12) to 24 (IQR 11–37.25).

**Table 1.** Number of days between request receipt and report transfer by 6-month period and in total.

	First 6 months	Second 6 months	Total 12 months
**Number of requests for information**	29	30	59
**Minimum time**	0	0	0
**25th percentile**	3	11	7
**Median time**	7	24	11
**75th percentile**	12	37	25
**Maximum time**	84	70	84
**Range**	84	70	84

**Figure 3:**
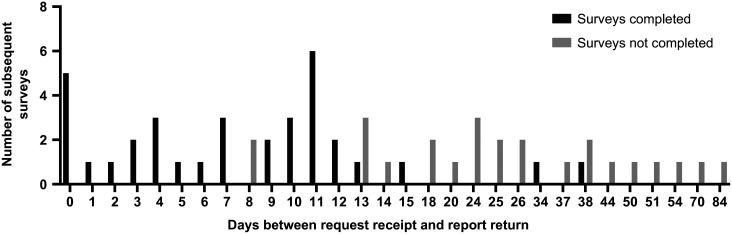
Figure 3. Number of completed and non-completed clinician questionnaires by delay in report completion.

**Figure 4:**
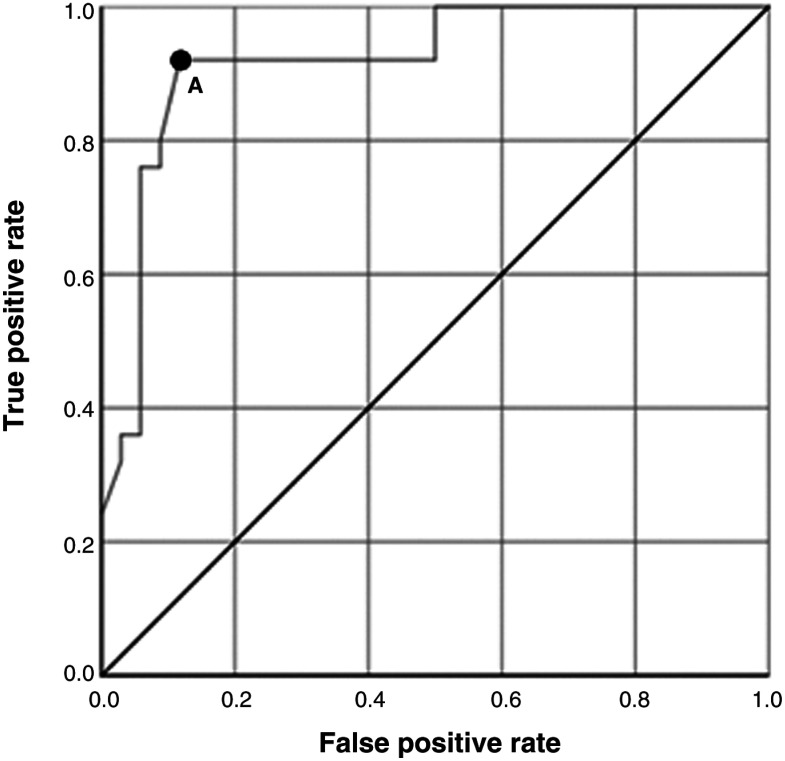
Figure 4. Receiver operating curve analysis of the number of days taken for the hospital to complete their report and return it to the pre-hospital team and the completion rates of the questionnaires by the pre-hospital teams after receiving the hospital’s report.

There was a median of five requests per month (IQR 2.25–7.75, range 1–9). LCs were completed in 86.4% (n = 51) of cases. Questionnaires were completed in 66.7% (n = 34) of cases in which LCs were conducted (Figure 2). Clinician questionnaires were completed and returned to the hospital team in 83.8% (n = 31) of cases where the report was returned to the educator in 14 days or less, but only 13.6% (n = 3) when reports took longer than 14 days (Table 2 and Figure 3). A ROC curve analysis demonstrated that the questionnaire response rate was poorer after 12 days rather than the empirically chosen ‘14-day target’ (Figure 4). A post-hoc analysis with Fisher’s exact test with a 95% CI demonstrated that there was a statistically significant difference in questionnaire completion rates between the first and latter six months, a drop of 86.2% (n = 25 of 29) to 30.0% (n = 9 of 30) (p < 0.0001). This may be associated with the reduction in 14-day performance between the two 6-month periods.

**Table 2.** Pre-hospital clinician questionnaire completion rates depending on whether the hospital report was made available to the facilitator within or outside of the 14-day target.

Delay between receipt of request and sending report	Questionnaire completed		Questionnaire not completed		Total
**≤ 14 days**	31	83.8%	6	16.2%	37
**> 14 days**	3	13.6%	19	86.4%	22
**Total**	34		25		59

Point A represents the threshold of 12 days or less for hospitals to complete the report. This was the number of days beyond which the rate of questionnaire completion by pre-hospital clinicians dropped substantially.

**Figure 5:**
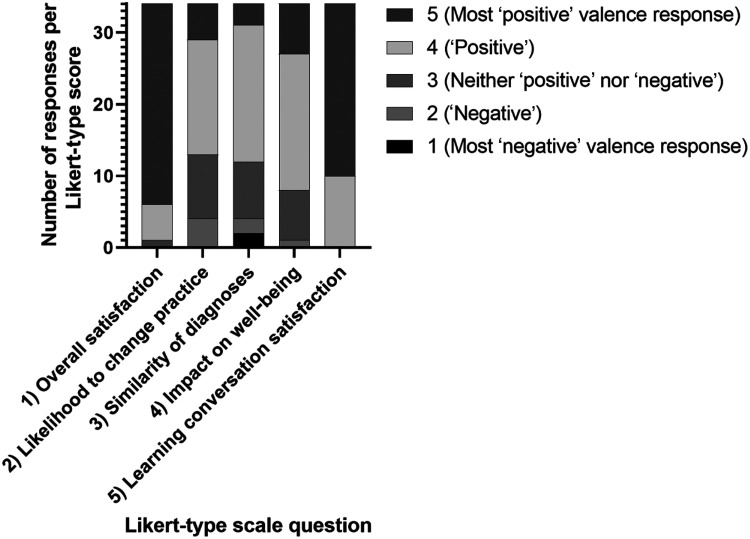
Figure 5. Frequency of Likert-type response scores per question.

Questionnaires were returned by pre-hospital clinicians in 34 cases (66.7% of those which had an LC conducted). Of the respondents, 82.4% (n = 28) cited being ‘very satisfied’ and 14.7% (n = 5) ‘satisfied’ with the information returned to them by the hospital team (Table 3 and Figure 5). Similarly, 70.6% (n = 24) were ‘very satisfied’ and 29.4% (n = 10) ‘satisfied’ with their LC.

**Table 3.** Absolute number and percentages of responses per Likert-type scale.

Question	Likert-type responses									
	Absolute number and percentages									
1. How satisfied are you with the returned information?	1 Very unsatisfied		2 Unsatisfied		3 Neither		4 Satisfied		5 Very satisfied	
	0	0.0%	0	0.0%	1	2.9%	5	14.7%	28	82.4%
2. How likely are you to change your practice in view of this information?	1 Very unlikely		2 Unlikely		3 Neither		4 Likely		5 Very likely	
	0	0.0%	4	11.8%	9	26.5%	16	47.1%	5	14.7%
3. How similar to your impression was the eventual diagnosis?	1 Very different		2 Different		3 Similar		4 Very similar		5 Identical	
	2	5.9%	2	5.9%	8	23.5%	19	55.9%	3	8.8%
4. Has this information helped your mental well-being?	1 Very adversely affected		2 Adversely affected		3 No impact		4 Positively affected		5 Very positively affected	
	0	0.0%	1	2.9%	7	20.6%	19	55.9%	7	20.6%
5. How satisfied are you with your debrief?	1 Very unsatisfied		2 Unsatisfied		3 Neither		4 Satisfied		5 Very satisfied	
	0	0.0%	0	0.0%	0	0.0%	10	29.4%	24	70.6%

Most pre-hospital clinicians reported that they planned to alter their practice in response to their interaction with the project (61.8% indicated they were either ‘likely’ (n = 16) or ‘very likely’ (n = 5) to, compared to 11.8% who indicated they were either ‘unlikely’ (n = 4) or ‘very unlikely’ (n = 0) to). The majority of pre-hospital clinicians also self-reported a high degree of similarity between their impression and the eventual hospital diagnosis. A total of 64.7% answered that their diagnosis was either ‘very similar’ (n = 19) or ‘identical’ (n = 3) to the diagnosis listed on the report from the hospital. ‘Positive’ (n = 19) or ‘very positive’ (n = 7) effects on mental health were reported by 76.5% of participants as a result of interaction with the project. Only 2.9% (n = 1) reported ‘adversely affected’ mental health. No respondents reported ‘very adversely affected’ mental health. No patients dissented and there were no data breaches.

## Discussion

All requests that met the inclusion criteria and followed the standard operating procedures received a report with legal support. However, the performance did not meet the empirical target of all reports being returned within 14 days. This voluntary team, working in their own time, were unable to sustain high performance across 12 months despite the low caseload. Dedicated time for the team within their employment duties may sustain performance.

The small pool of eligible clinicians at the included ambulance station may be responsible for limiting the numbers of requests (median five per month). Other projects have demonstrated higher mean numbers of cases ranging from 2.84 to 3.77 requests per week in conference abstracts (Collingwood et al., 2018; Jenkinson et al., 2009; Pollard & Black, 2015; Sommers et al., 2019) compared with a mean 1.13 per week in this system. All requests must be approved by an objective pre-hospital colleague. This additional step may dissuade some pre-hospital clinicians from engaging. Pre-hospital clinicians may also be deterred if they worry that facilitator involvement will lead to negative scrutiny of their practice. Delays and effort related to pre-hospital clinicians needing approval for NHSmail email addresses (a condition of the hospital’s information governance team) may also have deterred some potential participants.

Two of the longest delays in providing a hospital report (84 and 44 days) were due to the reluctance of a single clinician to request via a facilitator. The team were able to respond to these requests once the process was followed.

The reduction in ‘14-day performance’ between the first and last 6 months may have been influenced by the departure of the project lead (MS) from the hospital, who was then unable to access data and assist with reports. The efforts expended by the project lead may have been an example of the ‘Hawthorne effect’ and more than a typical team member. This should be taken into consideration when planning the team sizes. The remaining team rotated in line with medical training years. Most of the team did not work primarily within the ED. This introduced delays each time a request was received, as they had to travel down to the ED to access paper-based patient notes. This may be less of an issue in hospitals with electronic health records and where teams are embedded within the ED. The month-to-month variation in requests also made managing the hospital team and maintaining their engagement more challenging than might have been the case with a more consistent workload.

Doctors were used in the hospital team for the period of data collection. The authors believe that similarly motivated and experienced advanced clinical practitioners (particularly those with paramedic backgrounds), nurses and physician associates could also be considered.

The use of 14 days or less as the target for completion of requests appears reasonable. ROC analysis suggests 12 days or less may be more appropriate, albeit more challenging, based on this small sample.

There was no protected time to conduct LCs. Some of these were delayed due to one or both of the pre-hospital clinicians’ and facilitators’ leave periods and incompatible work schedules. Delays may have reduced the value of the learning experience and the likelihood that the pre-hospital clinician would put in further requests while awaiting the feedback. These limitations may mean that only the most motivated users engaged with the project, limiting overall numbers. Data on delays from report transfer to LC were not measured, meaning these delays cannot be quantified.

The minimum standard of content for reports for ambulance service staff was the hospital diagnosis. More comprehensive reports were provided for HEMS requests due to their lower frequency. Providing detailed reports to all ambulance service clinicians was an aspirational goal and this was consistently achieved as request volumes were lower than anticipated. These more nuanced and detailed reports could better focus on pre-hospital clinicians’ academic, professional and psychological needs. The most frequently cited reason for requesting information was due to ‘diagnostic uncertainty’ (80.7%). The addition of facilitators provides opportunities to establish the underlying factors of that uncertainty. This may be more effective for development than simply receiving the hospital diagnosis for comparison. The hospital team did highlight anecdotally that a greater number of requests would have necessitated less thorough reports for ambulance service clinicians as they felt they could provide little or no added time beyond what they were already committing.

The scrutiny of request legitimacy provided by facilitators and the use of NHSmail by all parties were important to demonstrate data security and achieve CAG support to conduct this service evaluation without consenting patients. To the authors’ knowledge, this is the first project to achieve this standard of recognition and medicolegal protection in an effort to provide HPHF for the purposes of clinician development, in a non-research setting and without patient consent. The absence of data breaches supports this approach.

No patients exercised their right to dissent. Patient-focused posters informing them of the project and their right to dissent were placed in the waiting rooms and corridors of the ED. Those who were critically unwell may not have had the awareness or opportunity to make note of these. The process had been approved by the hospital’s Patient Panel and a national patient charity, and has since been presented to multiple regional patient panels, with a positive reception. This suggests that, while falling short of a perfect way of informing patients, this is an acceptable use of patient data in the eyes of the public. More work is required to establish the opinions of the public, rather than their patient representatives, regarding HPHF.

The responses demonstrate high levels of satisfaction overall. In addition, every respondent cited that they were ‘satisfied’ or ‘very satisfied’ with their LC. This suggests that this ‘indirect’ model is received well by pre-hospital clinicians.

A single respondent indicated that the process adversely affected their mental well-being. Once identified, the authors contacted the respondent to learn more. The reason given was that there was no high-acuity problem identified by the hospital. This correlated with their impression but they were compelled by the patient to convey to ED. The pre-hospital clinician felt that they could have been directed to those in greater need of help, hence the negative response. This is an unexpected finding that did not lead to a poor satisfaction score.

The authors noted anecdotal comments from potential pre-hospital clinicians during the planning phase, who cited concerns that the use of senior colleagues as a standard part of the PHEM Feedback process was a covert attempt to identify practices which require disciplinary action. This may have dissuaded some from engaging. A reluctance for those who have experienced disciplinary procedures, blame or have previously received poorly delivered feedback to engage with the process may limit external validity in this cohort. The authors are of the opinion that it is important that HPHF should be used as part of a ‘just culture’ with adequate resources to meet gaps in the knowledge, skills and attitudes of participants. If such a culture can be established with authenticity and transparency, the authors hypothesise that others will feel safer and more empowered to follow what could be considered the ‘early adopter’ pre-hospital clinicians who participated.

### Limitations

There is the potential for selection bias in this sample of ambulance and HEMS clinicians, due to a 57.6% response rate overall. It is possible that those less satisfied with the service omitted the questionnaire if they were not interested in participating further.

LTSs were converted to numerical integers from 1 to 5 for analysis based on their valence (how ‘positive’ or ‘negative’ their response was). The scales were not all similar with regard to their valence. The question regarding similarity of diagnoses used ‘similar’ as the neutral option. Having ‘similar’, ‘very similar’ and ‘identical’ may have made it harder for respondents to select the most appropriate degree to which their diagnosis correlated with the hospitals. While each of the options on LTS were grammatically on a spectrum of valence, this introduces a potential for perceived, subjective judgement with regards to how positive or negative their performance was. For example, whether or not a clinician intends to change their practice does not necessarily mean either response reflects poorer or ‘more negative’ practice. The goal of the project was to provide more information to enhance professional development, which therefore can alter clinicians’ practice. A greater number of pre-hospital clinicians indicating that they would change their practice may suggest that the system was adding educational value, but value can also be derived from reinforcing good-quality care. The degree of similarity between the pre-hospital and hospital diagnoses also risks implying judgement. It is not reasonable to expect a pre-hospital working diagnosis to achieve the accuracy of a hospital diagnosis, gathered with the benefit of time, progression of the disease process and access to more diagnostic resources. This question was included to offer some insight into how frequently facilitators may encounter a situation where a pre-hospital diagnosis was markedly different to the diagnosis established in hospital, to inform development of training to support LCs in such cases. The use of subjective self-reported responses at this stage risks introducing biases based on the attitudes and experiences of the respondents to the question, for instance whether clinicians were prone to either cognitive dissonance or being very self-critical. A more objective approach would be a blinded chart review of the pre-hospital and hospital clinical records. It may also have been more valuable to focus on differences between the pre-hospital and hospital diagnoses which would have resulted in different decisions regarding treatment or destination hospital (such as a tertiary centre).

Some questions lacked clarity. The two questions assessing satisfaction were intended to distinguish the value added by the content of the hospital report and the value added by the facilitator with the LC. Satisfaction is a vague term and did not allow the respondent to specify which aspect of the information was helpful to their development, or in what way. The tool was not validated and was intended for local use to establish how HPHF could be sustained, rather than a peer-reviewed manuscript. Over time, the topic of HPHF has become more widely discussed and the authors felt that these data may be of use to the academic pre-hospital community, despite these limitations. A validated tool may mitigate these limitations.

Data were not collected with regards to the number of requests which were declined by the facilitator on the grounds of not meeting the inclusion criteria. One of the hypothesised benefits of including a facilitator was to filter requests, focusing the hospital team’s efforts on the timely delivery of fewer but higher-quality reports. Quantifying these exclusions may address this hypothesis.

## Conclusions

This small sample of participants using an ‘indirect’ HPHF model shows a majority of staff reporting high levels of satisfaction with both the returned information in reports and the LCs. The high levels of similarity between pre-hospital and hospital diagnoses reported among the study population is an encouraging finding, and suggests that HPHF should accommodate reinforcement of good practice, not just focus on discrepancies and areas for improvement. The majority of respondents cited a beneficial effect on their mental well-being following their engagement with the project.

The authors recommend that establishing reliable and medicolegally robust HPHF be considered as an objective for regulators, hospital trusts, ambulance services, HEMS organisations, commissioners and patient advocacy groups. The authors recommend that hospitals allocate resources beyond those described here in order to implement this work more sustainably. More work is required to determine the value that HPHF has on staff development and the causality between feedback and patient safety, and to quantify the costs to hospitals and ambulance services for staff performing the clinical reporting and facilitated feedback.

## Author contributions

MS devised the project, secured the section 251 non-research support, designed the standard operating procedures, contributed to the acquisition, analysis and interpretation of the data and contributed to all drafts of the manuscript.

GC and TW contributed to the acquisition, analysis and interpretation of the data, and contributed to all drafts of the manuscript.

GA contributed to the analysis and interpretation of the data, and contributed to all drafts of the manuscript.

All authors confirm that they approve this final version for publication and that they shall be accountable for all aspects of the work in ensuring that questions related to the accuracy or integrity of the work are appropriately investigated and resolved.

MS acts as the guarantor for this article.

## Conflict of interest

None declared.

## Ethics

Support was granted from the Health Research Authority Confidentiality Advice Group (CAG) (reference 18/CAG/0018).

## Funding

None.
